# The impact of hospital accreditation on quality measures: an interrupted time series analysis

**DOI:** 10.1186/s12913-015-0784-5

**Published:** 2015-04-03

**Authors:** Subashnie Devkaran, Patrick N O’Farrell

**Affiliations:** Cleveland Clinic Abu Dhabi, P.O. Box 112412, Abu Dhabi, United Arab Emirates; Edinburgh Business School, Heriot-Watt University, Riccarton, Edinburgh, EH14 4AS UK

**Keywords:** Accreditation, Joint Commission International, Healthcare quality measures, Interrupted time series analysis

## Abstract

**Background:**

Developing countries frequently use hospital accreditation to guarantee quality and patient safety. However, implementation of accreditation standards is demanding on organisations. Furthermore, the empirical literature on the benefits of accreditation is sparse and this is the first empirical interrupted time series analysis designed to examine the impact of healthcare accreditation on hospital quality measures.

**Methods:**

The study was conducted in a 150-bed multispecialty hospital in Abu Dhabi, United Arab Emirates. The quality performance outcomes were observed over a 48 month period. The quality performance differences were compared across monthly intervals between two time segments, 1 year pre- accreditation (2009) and 3 years post-accreditation (2010, 2011 and 2012) for the twenty-seven quality measures. The principal data source was a random sample of 12,000 patient records drawn from a population of 50,000 during the study period (January 2009 to December 2012). Each month (during the study period), a simple random sample of 24 percent of patient records was selected and audited, resulting in 324,000 observations. The measures (structure, process and outcome) are related to important dimensions of quality and patient safety.

**Results:**

The study findings showed that preparation for the accreditation survey results in significant improvement as 74% of the measures had a significant positive pre-accreditation slope. Accreditation had a larger significant negative effect (48% of measures) than a positive effect (4%) on the post accreditation slope of performance. Similarly, accreditation had a larger significant negative change in level (26%) than a positive change in level (7%) after the accreditation survey. Moreover, accreditation had no significant impact on 11 out of the 27 measures. However, there is residual benefit from accreditation three years later with performance maintained at approximately 90%, which is 20 percentage points higher than the baseline level in 2009.

**Conclusions:**

Although there is a transient drop in performance immediately after the survey, this study shows that the improvement achieved from accreditation is maintained during the three year accreditation cycle.

## Background

### Introduction

The frequency and magnitude of medical errors is gaining public focus [1]. In response to concerns about quality, escalating costs and government regulated accountability standards, healthcare leaders are seeking scientific methods for improving healthcare quality in hospitals. Optimal solutions, however, are proving to be elusive. Although several concepts, methodologies and tools have been postulated to advance quality and patient safety in healthcare [1-4], there still exists a dearth of compelling evidence of their impact and effectiveness, none more so than the all-encompassing strategy of accreditation [5-10].

Braithwaite, J. et al. [11] have argued that, “empirical evidence to sustain many claims about the benefits of accreditation is currently lacking”. Nevertheless, many countries, including the UAE, are frequently utilizing accreditation as a tool for government regulation to guarantee quality of care and improve patient safety. However, implementation of accreditation standards is demanding on individuals and organisations [12]. In addition, the theoretical and empirical literature on accreditation is sparse, especially in the emerging economies of the Middle East.

Previous research on the impact of healthcare accreditation shows inconsistent results [13,14]. Accordingly, there has been an extensive call in the healthcare literature for an assessment of such external accreditation systems to produce rigorous evaluations of their impact [9,15-18]. In this paper we report on an interrupted time series analysis of the impact of accreditation over a 4 year period (before and after accreditation) of a 150-bed hospital in Abu Dhabi.

### International accreditation

Joint Commission International (JCI) is a not-for-profit affiliate formed by The Joint Commission (TJC) to provide leadership in healthcare accreditation and quality improvement for organisations outside the United States. By 2013, JCI had accredited 500 healthcare organisations internationally [19]. A hospital seeking to obtain JCI accreditation is visited every three years by a survey team that observes hospital operations, conducts interviews, and reviews medical documentation for compliance with a set of standards. The goal of the survey is to evaluate care, organisational processes and to provide education with the objective of promoting continual improvement for the organisation under survey.

## Methods

The impact of accreditation has been researched adopting a variety of methodologies and research designs. There is a lack of rigorous research including the methodological challenges of measuring outcomes and attributing causality to these complex, changing, long-term social interventions to healthcare organisations [9]. Researchers have wrestled with a range of methodological issues, including research designs, selection bias, quality measurement, and the problems of evaluating outcomes. Most studies have used cross-sectional designs and/or comparative statistical analysis of data at two points in time [8,20-22]. Due to the dynamic nature of accreditation, such methodologies can only identify statistical associations between variables but cannot alone establish causality [23]. Instead, a dynamic statistical analysis technique is needed to draw causal inferences about the influence of accreditation on clinical quality, over time [9]. The use of a time series framework, in this research, directly addresses this issue. A longitudinal study enables causal relationships between variables to be determined. Furthermore, research projects that did demonstrate improvements in quality measures could not be generalized to acute care settings as they focused on a specific measures (e.g. AMI measures), types of services (e.g. cardiology) or organisations (e.g. teaching hospitals) [20-24]. This study is the first empirical interrupted time series analysis of accreditation designed to examine the impact of accreditation on hospital quality measures. No previous studies have used this methodology as it is difficult to maintain a controlled environment during the period of study. However the hospital analyzed did not undergo any significant organisational changes between 2009 and 2012. Thus both the leadership, organisational structure and the scope of services remained the same. Furthermore, the 27 quality measures selected reflect structures, processes and outcomes of care.

### Study design

Interrupted time series analysis, distinguishes between the effects of time from that of the intervention and is the most powerful, quasi-experimental design to evaluate longitudinal effects of such time-limited interventions [25,26]. The interruption splits the time series into pre-intervention and post- intervention (accreditation) segments so that segmented regression analysis of interrupted time series data permits the researcher to statistically evaluate the impact of an intervention on an outcome variable, both immediately and long-term; and the extent to which factors other than the intervention explain the change. The choice of the beginning (2009) and end of each segment (2012) is linked to the start of the intervention (JCI accreditation occurred in December 2009). In this study, two parameters were used to define each segment of the time series: level and trend. The level is the value of the series at the beginning of a given time interval (i.e. the Y intercept for the first segment, and the value immediately following a change point or intervention). The trend is the rate of change of a variable (the slope) during a segment. Segmented regression analysis enables identification of the level and trend in the pre-accreditation (pre-intervention) segment and changes in level and trend after accreditation (post-intervention).

### Study population

The study was conducted in the private 150-bed, multispecialty, acute care hospital in Abu Dhabi, UAE. The annual in-patient census was 15,000. The hospital treats approximately half a million ambulatory care patients per year. The scope of healthcare services is provided to all patient age groups, nationalities and payment types.

### Data source and study variables for clinical quality measures

The outcome measures for the time series analysis incorporated clinical quality measures, including mortality rates etc. and were expressed as percentages, proportions or rates. These performance differences were compared across monthly intervals between two time segments, 1 year pre-accreditation (2009) and 3 years post-accreditation (2010, 2011 and 2012) for the selected quality measures (Table 1). The principal data source was a random sample of 12,000 patient records drawn from a population of 50,000 during the study period (January 2009 to December 2012), resulting in 324,000 observations /data points. Slovin’s formula was used to calculate the sample size per month based on a 95% confidence interval from an average monthly in-patient census of 1,500 patients. Each month (during the entire study period), a simple random sample of 24% of patient records were selected and audited from the monthly population.

**Table 1:** **Quality measure descriptions**

**Dimension of measurement**		**Measures**	**Value**
Patient Assessment	Y_1_	Initial medical assessment done within 24 hours of admission	Percentage
	Y_2_	Initial nursing assessment within 24 hr. of admission	Percentage
	Y_3_	Pain assessment form completed 100% per month	Percentage
	Y_4_	Percentage of completed pain reassessment	Percentage
Laboratory Safety	Y_5_	Monitor the timeliness of complete blood count (cbc) as routine lab results	(in hours)
	Y_6_	The turnaround time of troponin lab results	(in minutes)
Surgical Procedures	Y_7_	Completion of surgical invasive procedure consent	Percentage
	Y_8_	Percentage of operating room (or) cancellation of elective surgery	Percentage
	Y_9_	Unplanned return to OR within 48 hours	Percentage
Medication error use and near-misses	Y_10_	Reported medication error	Per 1000 prescriptions
Anaesthesia and Sedation Use	Y_11_	Completed anaesthesia, moderate and deep sedation consent forms	Percentage
	Y_12_	Completed Modified Aldrete Scores (Pre, Post, Discharge)	Percentage
	Y_13_	Completed pre-anaesthesia assessments	Percentage
	Y_14_	Completion of anaesthesia care plan	Percentage
	Y_15_	Percentage of completed assessment of patient who received anaesthesia	Percentage
	Y_16_	Effective communication of risk, benefit and alternatives of anaesthesia explained to patients	Percentage
Availability, Content and Use of Patient Records	Y_17_	Percentage of typed post-operative report completed with 48 hours	Percentage
Infection Control, Surveillance and Reporting	Y_18_	Hospital acquired methicillin resistant staph aureus (MRSA) rate	Per 1000 Admissions
	Y_19_	Healthcare associated infection hospital-wide	per 1000 patient days
	Y_20_	Surgical site infection rate	Percentage
Reporting of Activities as Required by Law and Regulation	Y_21_	Mortality rate	Percentage
International Patient Safety Goals	Y_22_	Monitoring correct site marking	Percentage
	Y_23_	Monitoring compliance with the time-out procedure	Percentage
	Y_24_	Screening of patient fall risk	Percentage
	Y_25_	Overall hospital hand hygiene compliance rate	Percentage
	Y_26_	Patient fall rate	Per 1000 patient days
	Y_27_	Fall risk assessment and reassessment	Percentage

Source: Subashnie Devkaran.

The first criterion for measure selection was that all variables must be directly linked to a JCI standard. Second, the measures should reflect high priority areas that will affect outcomes of care. Third, the measures have a pre-defined profile which is based on: the process, procedure, or outcome to be measured; the availability of science or evidence supporting the measure; the dimension of quality that is captured by the measure, e.g. timeliness etc.; and the frequency of measurement. Finally, all measures are applicable to all patients in the hospital and are not specific to a specialty or disease. An internal data validation process is in place within the hospital included: re-collecting the data by second person not involved in the original data collection; using a statistically valid sample of records, cases or other data; comparing the original data with the re-collected data; calculating the accuracy by dividing the number of data elements found to be same by the total number of data elements and multiplying that total by 100. A 90% accuracy level is considered an acceptable benchmark. When the data elements differed, the reasons were noted (for example, unclear data definitions) and corrective actions were taken. A new sample was collected after all corrective actions have been implemented to ensure the actions resulted in the desired accuracy level.

### Ethics statement

The study was approved by the AN Hospital Ethics Committee. Furthermore, no identifiable human data were used for this study. The anonymous data set in the study was only accessible to the researchers thus, patient privacy was ensured.

### Data analysis of the clinical quality measures

#### Interrupted time series analysis

Segmented regression models fit a least squares regression line to each segment of the independent variable, time, and thus assume a linear relationship between time and the outcome within each segment [27]. The following linear regression model is specified to estimate the level and trend in the dependent variable before accreditation and the changes in level and trend following accreditation.

$$ {Y}_t={\upbeta}_0+{\upbeta}_1*\ tim{e}_t+{\upbeta}_2*\  interventio{n}_t+{\upbeta}_3*\  time\  after\  interventio{n}_t + {e}_t $$

Where, *Y*_*t*_ is the outcome, time *t* indicates time in months at *time*_t_ from the start of the observation period to the last time point in series; *intervention* is a measure for *time*_t_ designated as a dummy variable taking the values 0 occurring before intervention and 1 after the intervention (accreditation), which was implemented at month 12 in the series; *time after intervention* is a continuous variable recording the number of months after the intervention at *time*_*t*_, coded 0 before the accreditation and (time-36) after the accreditation. In this model:

- β_0_ is the baseline level of the outcome at the beginning of the series;
- β_1_ is the slope prior to accreditation (i.e. the baseline trend);
- β_2_ is the change in level immediately after the accreditation;
- β_3_ is the change in the slope from pre to post-accreditation; the sum of β_1_ and β_3_ is the post-intervention slope and
- *e*_*t*_ represents the random error term.

There are three particular characteristics of time-series— auto-correlation, non-stationarity, and seasonality which may lead to biased results [28]. The solutions to these problems are outlined below.

#### Autocorrelation, non-stationarity and seasonality in time series

If the Durbin-Watson statistic for first-order autocorrelation is significant, the model is adjusted by estimating the autocorrelation parameter and including it in the segmented regression model. Second, in order to establish whether a given time series displays autocorrelation, it is necessary to first render that series stationary. Non-stationarity relates to the data exhibiting one or more natural trends, implying that the mean value and variance of the data series can change over time for reasons exclusive of the effect of the intervention [29]. Finally, seasonality needs to be controlled since the pre-accreditation and post-accreditation time periods contain different seasonal profiles (e.g. more summer months in the post-accreditation period), which could potentially distort the actual effect of an intervention [28]. If the series displays seasonality or some other non-stationary pattern, the usual solution is to take the difference of the series from one period to the next and then analyze this differenced series. Sometimes a series may need to be differenced more than once or differenced at lags greater than one period. In order for seasonal autocorrelation terms to be identified and estimated, it is necessary that the series does not contain a seasonal unit root. Formal statistical testing for the presence of unit roots in time series was conducted using the Dickey-Fuller Test [30]. The series is stationary/no seasonality if P < 0.05.

In order to render a series stationary, obtaining a constant mean level of a series is achieved by removing any apparent trend component contained in this series. There are two general approaches to achieving this: (1) differencing the series by subtracting from each time point t_1_ the value of the previous time point t-1 or (2) de-trending the series using a regression approach and working with the model residuals. The Dickey-Fuller statistic [30] is used to test for stationarity. In cases where the null hypothesis of a unit root was rejected under this model, it was assumed that the series did not require differencing. Where the null hypothesis of a unit root is not rejected, then further analysis was done before concluding that differencing is required. This study uses a 48 month time series from the period January 2009 to December 2012, sufficient to enable detection and modelling of any seasonal patterns [31].

## Results

### Patient assessment measures

Table 2 displays the segmented regression equations of the time series before and after accreditation for the dependent variables of Initial Medical Assessments *(Y*_*1*_*)*, Initial Nursing Assessments *(Y*_*2*_*)*, Pain Assessments *(Y*_*3*_*)*, and Pain Reassessments *(Y*_*4*_*)*. Accreditation did not have a significant positive impact on the assessment quality measures of *Y*_*1*_, *Y*_*2*_ and *Y*_*3*_. Hospitals are mandated to publish a four month track record of compliance prior or accreditation (Joint Commission International, 2010) and thus the results may be influenced in part by the high compliance with the standard prior to the accreditation survey. Furthermore, only one of the measures (percentage of completed pain reassessments) had a significant decrease in the slope post accreditation survey. It also recorded a significant pre-accreditation slope.

**Table 2:** **Patient assessment and laboratory safety measures**

**Model validation and parameter estimation**												**Diagnostic tests**	
**Patient assessment measures**	**Intercept**		**Time(β**_**1**_ **)**		**Intervention (β**_**2**_ **) (Change in level)**		**Tim_Aft_Int (β**_**3**_ **) (Change in slope)**		**R** ^**2**^	**Autocorrelation (AC) Check**		**Test for Seasonality/Stationarity**	
										**(Durbin Watson)**		**(Dickey Fuller Unit Root Test)**	
	**Value**	**P-value**	**Value**	**P-value**	**Coefficient 95% confidence interval (LCI-UCI)**	**P-value**	**Coefficient 95% confidence interval (LCI-UCI)**	**P-value**	**R** ^**2**^	**D-Value (before)**	**D-Value (after)**	**P-value**	**Result**
***Y***_***1***_	78.60	0.00*	1.19	0.35	−4.54 (−16.33 to 7.25)	0.44	−0.99(−3.63 to 1.65)	0.45	0.38	1.00	1.92	0.03	No Seasonality
***Y***_***2***_	96.17	0.00*	0.13	0.53	1.24 (−1.63 to 4.11)	0.38	−0.18 (−0.60 to 0.24)	0.39	0.09	1.46	No AC	0.00	No Seasonality
***Y***_***3***_	94.56	0.00*	0.16	0.85	−4.00 (−12.10 to 4.10)	0.33	−0.02 (−1.82 to 1.77)	0.98	0.34	1.05	2.22	0.04	No Seasonality
***Y***_***4***_	32.56	0.00*	7.02	0.00*	−13.91 (−32.37 to 4.56)	0.14	−7.28 (−10.00 to −4.56)	0.00*	0.48	1.72	No AC	0.00	No Seasonality
**Laboratory safety measures**													
***Y***_***5***_ **(in hours)**	7.06	0.00*	−0.36	0.00*	0.34(0.13, 0.54)	0.52	0.34(0.04, 0.64)	0.00*	0.73	1.31	2.11	0.04	No Seasonality
***Y***_***6***_ **(minutes)**	47.58	0.00*	0.15	0.46	−0.43(−2.99, 2.13)	0.74	−0.60(−1.02, −0.18)	0.01*	0.90	1.85	No AC	0.95	Data is not stationary

AC refers to Autocorrelation, D-Value is the Durbin Watson statistic, *P ≤ 0.05 is considered significant.

### Laboratory safety measures

The outcome of analysis for the segmented regression analysis for Timeliness of Complete Blood Count as a Routine Lab Result (in hours) *(Y*_*5*_*)* and turnaround time of Troponin Lab Results (minutes) *(Y*_*6*_*)* (Table 2) demonstrated different results. The increase in *Y*_*5*_ measure (turnaround time) immediately post- accreditation was not significant but had a significant positive change in the slope (P ≤ 0.0001) pre-accreditation and post-accreditation. Conversely, the *Y*_*6*_ measure (turnaround time) decreased immediately post- accreditation survey with a significant negative change in slope (P ≤ 0.0001). The positive *Y*_*6*_ measure results may be explained by the demand for the laboratory results by the Emergency Department, a process independent from accreditation. In addition, the implementation of a clinical pathway on Acute Myocardial Infarction requires the laboratory to improve the turnaround time for Troponin as it is an important decision making tool for clinicians.

### Surgical procedures

There is a significant change in the level of the *Y*_*7*_ measure (surgical procedure consent) after accreditation (P ≤ 0.01) followed by a significant decrease in slope (Table 3). The results may be attributed to the relatively high pre-accreditation performance. Conversely, accreditation had no significant impact on the operating room measures *Y*_*8*_ (percentage cancellations of elective surgery) and *Y*_*9*_ (percentage return in OR within 48 hours).

**Table 3:** **Surgical procedures and medication error use and near-misses**

**Model validation and parameter estimation**												**Diagnostic tests**	
**Surgical Procedure Measures**	**Intercept**		**Time(β**_***1***_ **)**		**Intervention (β**_***2***_ **) (Change in level)**		**Tim_Aft_Int (β**_***3***_ **) (Change in slope)**		**R** ^**2**^	**Autocorrelation (AC) Check (Durbin Watson)**		**Test for Seasonality/Stationarity (Dickey Fuller Unit Root Test)**	
	**Value**	**P-value**	**Value**	**P-value**	**Coefficient 95%Confidence Interval (LCI, UCI)**	**P-value**	**Coefficient 95%Confidence Interval (LCI, UCI)**	**P-value**		**D-Value (before)**	**D-Value (after)**	**P-value**	**Result**
***Y***_***7***_	87.91	0.00*	1.21	0.00*	−2.70(−4.76, −0.63)	0.01*	−1.18(−1.72, −0.64)	0.01*	0.96	1.30	2.53	0.00	No Seasonality
***Y***_***8***_	14.89	0.00*	−0.28	0.38	−0.36(−4.66, 3.95)	0.87	0.32(−0.31, 0.95)	0.31	0.49	2.10	No AC	0.00	No Seasonality
***Y***_***9***_	0.08	0.5	0.003	0.88	−0.05(−0.30, 0.20)	0.69	0.01(−0.03, 0.04)	0.63	0.34	1.86	No AC	0.00	No Seasonality
**Reported medication error measure**													
***Y***_***10***_	0.03	0.03*	0.002	0.21	−0.04(−0.06, −0.01)	0.00*	−0.00(−0.01, 0.00)	0.18	0.35	1.56	No AC	0.00	No Seasonality

AC refers to Autocorrelation, D-Value is the Durbin Watson statistic, *P ≤ 0.05 is considered significant.

### Reported medication errors

The results in Table 3 demonstrate that immediately following the accreditation survey, the level of reported medication errors per 1000 prescriptions (*Y*_*10*_*)* dropped significantly (P ≤ 0.001), but there was no significant change in the slope after the intervention. A quality improvement project to reduce the number of medication errors had been implemented in September 2009 (3 months before the survey). Moreover, the JCI survey has a comprehensive approach (medication system tracer) to evaluate compliance which may have led to the significant improvement. However, this improvement was not sustained.

### Anesthesia and sedation measures

The accreditation survey was followed by a negative change in level for five out of six measures, anesthesia and sedation measures (*Y*_*11,*_*Y*_*12,*_*Y*_*14,*_*Y*_*15*_ and *Y*_*16*_), excluding*Y*_*13*_, of which four (*Y*_*11,*_*Y*_*12,*_*Y*_*14,*_ and *Y*_*16*_) were significant (P ≤ 0.01) (Table 4). Similarly, all six anesthesia measures demonstrated a negative change in slope post-survey of which four (*Y*_*11,*_*Y*_*12,*_*Y*_*14,*_ and *Y*_*16*_) were significant (P ≤ 0.01). The negative change in post-accreditation slope is mainly due to staff not sustaining the improvement, as there was no incentive to do so due to the three year survey cycle.

**Table 4:** **Anesthesia and sedation use measures**

**Model validation and parameter estimation**												**Diagnostic tests**	
**Anesthesia and Sedation Use Measures**	**Intercept**		**Time(β**_***1***_ **)**		**Intervention (β**_***2***_ **) (Change in level)**		**Tim_Aft_Int (β**_***3***_ **) (Change in slope)**		**R** ^**2**^	**Autocorrelation (AC) Check (Durbin Watson)**		**Test for Seasonality/Stationarity (Dickey Fuller Unit Root Test)**	
	**Value**	**P-value**	**Value**	**P-value**	**Coefficient 95%Confidence Interval (LCI, UCI)**	**P-value**	**Coefficient 95%Confidence Interval (LCI, UCI)**	**P-value**		**D-Value (before)**	**D-Value (after)**	**P-value**	**Result**
***Y***_***11***_	55.19	0.00*	5.02	0.00*	−15.42(−23.38, −7.45)	0.00*	−4.95(−6.12, −3.78)	0.00*	0.71	1.84	No AC	0.00	No Seasonality
***Y***_***12***_	28.87	0.00*	7.2	0.00*	−7.17(−12.11, −2.23)	0.01*	−7.30(−8.49, −6.11)	0.00*	0.81	2.84	1.91	1.00	Data is not Stationary
***Y***_***13***_	92.15	0.000*	0.7	0.22	0.97(−4.86, 6.80)	0.74	−0.84(−1.98, 0.30)	0.14	0.33	1.27	1.91	0.02	No Seasonality
***Y***_***14***_	77.43	0.00*	2.61	0.00*	−11.68(−20.04, −3.31)	0.01*	−2.48(−4.07, −0.88)	0.00*	0.8	0.78	2.13	0.00	No Seasonality
***Y***_***15***_	97.01	0.000*	0.22	0.81	−6.17(−14.37, 2.03)	0.14	−0.02(−1.90, 1.87)	0.98	0.45	0.92	1.75	0.00	No Seasonality
***Y***_***16***_	67.2	0.00*	3.75	0.00*	−12.83(−21.63, −4.03)	0.01*	−3.64(−4.94, −2.35)	0.00*	0.53	1.76	No AC	0.00	No Seasonality

AC refers to Autocorrelation, D-Value is the Durbin Watson statistic, *P ≤ 0.05 is considered significant.

### Completion of the typed post-operative note within 48 hours

The results in Table 5 demonstrate an increase in the level of *Y*_*17*_ measure but this was not significant. Conversely, the negative post-accreditation slope is significant (P ≤ 0.01). These results reveal that improvement was not sustained after accreditation, which may be due to the relatively high existing compliance.

**Table 5:** **Infection control and content and use of patient records measures**

**Infection Control, Surveillance and Reporting Measures**	**Intercept**		**Time(β**_***1***_ **)**		**Intervention (β**_***2***_ **) (Change in level)**		**Tim_Aft_Int (β**_***3***_ **) (Change in Slope)**		**R** ^**2**^	**Autocorrelation (AC) Check (Durbin Watson)**		**Test for Seasonality/Stationarity (Dickey Fuller Unit Root Test)**	
	**Value**	**P-value**	**Value**	**P-value**	**Coefficient 95%Confidence Interval (LCI, UCI)**	**P-value**	**Coefficient 95%Confidence Interval (LCI, UCI)**	**P-value**		**D-Value (before)**	**D-Value (after)**	**P-value**	**Result**
***Y***_***18***_	6.90	0.00*	-0.71	0.00*	1.41(0.09, 2.72)	0.04*	0.70(0.31, 1.100	0.001*	0.30	1.63	No AC	0.00	No Seasonality
***Y***_***19***_	0.65	0.22	-0.05	0.48	0.25(-0.81, 1.32)	0.63	0.08(-0.08, 0.23)	0.33	0.12	1.61	No AC	0.00	No Seasonality
***Y***_***20***_	0.08	0.50	0.00	1.00	-0.05(-0.29, 0.18)	0.64	0.00(-0.03, 0.04)	0.81	0.51	2.31	No AC	0.00	No Seasonality
**Availability, Content and Use of Patient Records**													
***Y***_***17***_	57.33	0.000*	1.95	0.0058*	4.33(-4.98, 13.64)	0.35	-1.85(-3.22, -0.480	0.01*	0.54	1.75	No AC	0.01	No Seasonality
**Mortality Rate**													
***Y***_***21***_	-0.04	0.59	0.02	0.15	-0.01(-0.16, 0.140	0.90	-0.02(-0.04, 0.01)	0.15	0.10	2	No AC	0.00	No Seasonality

AC refers to Autocorrelation, D-Value is the Durbin Watson statistic, *P ≤ 0.05 is considered significant.

### The infection control measures

Following the accreditation survey, the level of two out of the three infection control measures increased (excluding *Y*_*20)*_ of which *Y*_*18*_ was significant (*Y*_*18)*_, (P ≤ 0.05) (Table 5). However all three measures exhibit an increase in the slope post- survey of which *Y*_*18*_ is significant (P ≤ 0.05). This may be partly attributed to a more developed infection control programme and surveillance process after the survey, thus resulting in the identification of more infections.

### Mortality rate

None of the coefficients for mortality rate *Y*_*21*_ is significant (Table 5). This is largely due to the fact that the JCI standards are more process and structure oriented and thus would not impact on outcome measures. The standards do not address clinical care at a physician or practice level.

### International Patient Safety Goal (IPSG) Measures

Four out of the six patient safety goal measures recorded an immediate decrease in level post-accreditation survey, but only (*Y*_*23*_*)* was significant (Table 6). While five out of the six measures recorded a negative change in the post- accreditation slope, of which four (*Y*_*22,*_*Y*_*23,*_*Y*_*24*_ and *Y*_*27*_) were significant. The purpose of the IPSGs is to highlight problematic areas in health care and to promote specific improvements in patient safety. These measures are important to the organisation and thus the pre-accreditation and overall performance was relatively high. In addition, both the accreditation survey and implementation of the standards did not have a significant effect as the organisation had already implemented the safe surgery practice prior to these interventions.

**Table 6:** **International patient safety goals**

**Model validation and parameter estimation**												**Diagnostic tests**	
**International Patient Safety Goal Measures**	**Intercept**		**Time(β**_***1***_ **)**		**Intervention (β**_***2***_ **) (Change in Level)**		**Tim_Aft_Int (β**_***3***_ **) (Change in Slope)**		**R** ^**2**^	**Autocorrelation (AC) Check (Durbin Watson)**		**Test for Seasonality/Stationarity (Dickey Fuller Unit Root Test)**	
	**Value**	**P-value**	**Value**	**P-value**	**Coefficient 95%Confidence Interval (LCI, UCI)**	**P-value**	**Coefficient 95%Confidence Interval (LCI, UCI)**	**P-value**		**D-Value (before)**	**D-Value (after)**	**P-value**	**Result**
***Y***_***22***_	40.56	0.000*	5.20	0.00*	0.79(−4.37, 5.94)	0.76	−5.269-6.19, −4.34)	0.00*	0.94	1.05	2.07	0.00	No Seasonality
***Y***_***23***_	25.70	0.000*	7.51	0.00*	−14.89(−21.30, −8.49)	0.00*	−7.36(−8.64, −6.08)	0.00*	0.90	1.1	2.43	0.14	Seasonality
***Y***_***24***_	91.94	0.000*	0.65	0.00*	0.21(−2.46, 2.89)	0.87	−0.67(−1.07, −0.28)	0.00*	0.42	1.96	No AC	0.00	No Seasonality
***Y***_***25***_	−0.02	0.96	0.02	0.71	0.14(−0.43, 0.71)	0.62	−0.02(−0.11, 0.06)	0.62	0.03	1.72	No AC	0.00	No Seasonality
***Y***_***26***_	98.48	0.00*	−0.10	0.1	1.71(1.04, 2.38)	0.00*	0.11(0.00, 0.230)	0.06	0.52	2.86	2.03	0.06	Data Not Stationary
***Y***_***27***_	55.51	0.00*	55.51	0.00*	−1.67(−6.29, 2.96)	0.47	−4.26(−5.30, −3.22)	0.00*	0.90	0.89	2.6	0.26	No Seasonality

AC refers to Autocorrelation, D-Value is the Durbin Watson statistic, *P ≤ 0.05 is considered significant.

The above effects may be attributed to three factors. First, surgical safety was considered an organisational priority and thus a Failure Modes Effects Analysis (FMEA) was conducted as a quality improvement project. This required that the surgical team review the surgical safety process and the potential areas of failure. An action plan was formulated to circumvent error prone processes and the JCI Universal protocol for safe surgery was implemented in July 2009. Second, JCI considers surgical safety and the universal protocol as an International Patient Safety Goal. Organisations that fail this standard, fail the entire accreditation survey. Finally, surgery on the incorrect patient, site or side is known as a sentinel event. The repercussions for the organisation are serious and mandate reporting to JCI and HAAD, which, may result in unfavorable publicity that would adversely affect the reputation of the hospital. Most importantly, wrong site surgery may cause permanent harm or death in a patient.

### Impact of the accreditation survey (December 2009) on the 27 quality measures

1. From the analysis, 20 of the 27 (74%) measures display a positive pre-accreditation slope of which thirteen (48%) are statistically significant (P ≤ 0.05).
2. A key finding is that accreditation had no significant impact (either positive or negative) on 11 out of the 27 measures.
3. The accreditation survey resulted in a significant positive change in level for only 2 (7%) of the measures (medication errors and hand hygiene compliance). Conversely, a significant negative change in level was observed in 7 (26%) of the measures.
4. Only 1 measure (4%), (Troponin turnaround time) resulted in a significant positive change in the post-accreditation slope.
5. Accreditation was associated with a significant negative change in slope in 13 (48%) of the measures.
6. Of the 27 quality measures, there is no significant positive change in the level of 25 measures post-accreditation. Additionally, there was no significant positive change in the slope of 26 measures post-accreditation.

## Discussion

Accreditation resulted more frequently in a significant negative change in level (7 measures) than a positive change in level (2 measures) after the survey. Moreover, accreditation had a much larger significant negative effect (48% of measures) than a positive effect (4%) on the slope. Even though the organisation had no significant changes in structure or service lines, and the same Quality Manager was employed for the entire period of observation, accreditation improvement proved difficult to sustain. Continuous survey readiness is fundamental and thus a policy of unannounced surveys may well enhance performance improvement. Frequent internal or external surveys may also encourage organisations to maintain the process of improvement. In addition, since many of the measures had existing high values pre-accreditation, any improvement in the performance may have been too small to be statistically significant.

**Figure 1:**
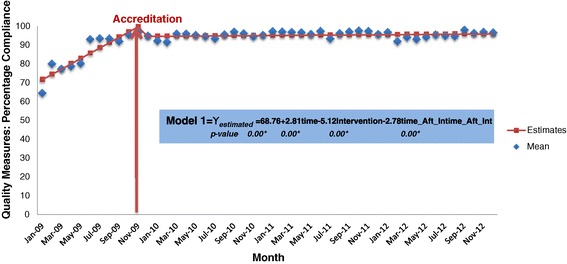
**Time Series Graph of the percentage of compliance before and after the accreditation survey. Note:** the average means of the following quality measures were used to create this time series graph: Y_1,_ Y_2,_ Y_3,_ Y_4,_ Y_7_, Y_11_- Y_17_, Y_22_- Y_25,_ Y_27_. The red arrow demarcates the Accreditation survey which occurred in December 2009.

Figure 1 illustrates the pattern of accreditation compliance using quality measures. The hospital ramps up its performance prior to the survey. There is a sharp incline in the pre-accreditation slope with an immediate drop post-accreditation survey. This is followed by an undulating plateau in performance during the three year period. The results demonstrate that once the accreditation survey is finished and the surveyors have left the organisation, performance plateaus. However, the figure shows that there is residual benefit from accreditation three years later with performance maintained at approximately 90%, which is 20 percentage points higher than the baseline level in 2009.

It can be argued that the on-site evaluation during an accreditation survey might only be seen as an organisational snapshot on the given day of the survey and thus all accreditation systems suffer from the potential criticism that their impact ends following completion of the survey. In order to sustain their value, there is a need to encourage participants to continue to maintain perceive benefits from the standards. This is not only in line with the models of continuous quality improvement; it also makes good commercial sense [32]. Limited life expectancy of the accreditation status is a way to deal with this. It can be argued that the standards are not ‘sensitive’ enough to allow the possibility of actually evaluating improvements. This is based on the fact that it has been found by other accreditation organisations that several institutions already comply with the accreditation standards the first time around, and therefore based on the way that the standards are formulated, an improvement of quality by an organisations does not necessarily lead to receiving a higher degree of compliance of the standards because the organisation has already fully complied with them. This is largely because the standards are maximum achievable across all types of hospitals independent of their complexity and service lines. In addition, the pass/fail concept does not drive performance beyond achieving compliance with standards. Thus excellent organisations that already comply with the standards are not incentivized by the accreditation process to improve their level of performance. So, although a thorough accreditation survey is designed to help draw conclusions about the overall quality and capability of an organisation, it is important to recognize that this triennial snapshot is no substitute for ongoing monitoring. Strategies are as a result required to reinforce the way accreditation might lead to improved quality of care. In recent times, alternative approaches used by The Joint Commission, in the United States, such as unannounced surveys and tracking patients with tracer methodologies along their path through a healthcare organisation, from pre-entry to discharge, are designed to help bring about improvements in accreditation processes and organisational and clinical systems, but these are all relatively untested [33].

### Recommendations

Benchmarking of accredited organisations’ by the accrediting body and submission of quality measures to a data library will ensure improvement between surveys. At the time of writing, JCI does not have a data library for benchmarking of accredited organisations. Benchmarking allows sharing of best practices and holds organisations accountable for maintaining good performance. Creating a library of mandatory reporting measures that are shared publicly or with other internationally accredited organisations would improve performance [34]. In recent times, healthcare organisations have begun focusing on the measurement of clinical effectiveness. Thus, in order demonstrate the efficacy of treatments, appropriate outcome measures were sought to assist in policy and management decisions about the appropriateness and the selection of clinical treatment. In addition, reliable and valid clinical outcome measures would answer the fundamental question of whether accreditation and compliance to its standards, has a causal relationship with patient outcomes.

JCI has no intra-cycle survey or periodic assessments between the survey periods. The Joint Commission in the US has established the expectation of continual readiness with the implementation of the unannounced survey in 2009. The Periodic Performance Review is also a Joint Commission annual requirement. Organisations assess their level of compliance for each standard and element of performance. This self-assessment forms the basis of the improvement efforts for gaps in compliance [35]. However, these self-assessments are not mandated by JCI. It is recommended that there should be a shift in the accreditation inspection process from a scheduled to unscheduled survey which will result in a change from a survey preparation mindset to that of continual readiness.

The accreditation standards largely review processes of care and not clinical outcomes. A crucial issue with the choice of implementing an accreditation model is ultimately whether accreditation even ensures quality, or has positive effects on the quality of care delivered by the accredited organisations. Achieving accreditation is typically regarded as a predictor of clinical care and organisational effectiveness by funders, institutions, patients and the public. This is meant to create confidence in the quality of care provided by an organisation. However, there is no real guarantee that an organisation which is well assessed during the accreditation process will always provide high quality care [36]. Accreditation only guarantees that the organisation meets standards which are deemed necessary by the accreditation organisation. Thus, although we are living in an increasingly evidence-based world, there has been little concrete evidence about the impact that individual accreditation programmes have on the healthcare system, healthcare providers and other stakeholders [5]. Similar to this study, there is evidence that hospitals rapidly increase compliance with the accreditation standards and improve their organisational processes in the months prior to the surveys, but there is still much less evidence that this brings benefits to the clinical process and the outcome of the healthcare systems [5,37].

The findings of this study should be read in light of its limitations. The study is based on a single hospital. However, such an approach has one major advantage: focusing upon one hospital provides a controlled environment, which is necessary for the time series methodology and permitted the researchers to attribute changes in the quality measures to the intervention of accreditation. Additionally, the use of primary data, the large sample size (over 12,000 patient records were reviewed), the variation and large number of quality measures (27 measures covering various dimensions of quality), the length of the study period and the number of observations (324,000 observations) compensates for this limitation. The study is set in resource- rich UAE and thus cannot be compared to developing countries. Nonetheless, the study evaluated international accreditation, which is a voluntary process and applied in many parts of the world. The researchers recommend that the validity of this study is tested in other settings. Only 7 out of the 27 measures were outcome measures. This is primarily because the study objective was to evaluate compliance and thus measures were linked to a specific standard. The challenge with the inclusion of outcome measures is to isolate the change in the measure due to accreditation and not for example, the disease process, comorbidities, the competence of the healthcare professional and other contributing factors. Further research is recommended to determine the impact of accreditation on patient outcomes.

## Conclusion

The most commonly used approach to evaluating accreditation systems has been a perception of benefits approach, which allows individuals to record their interpretations of improvements in the quality of service, changes in practices and their satisfaction with the process. Although perceived benefits are important in determining the acceptability of the accreditation process, they do not demonstrate that any change has taken place in the delivery of healthcare [32]. Whilst many postulations about the benefits of accreditation processes exist, empirical evidence to prove those claims is still currently lacking. According to Greenfield and Braithwaite [18] the fact that the empirical evidence base for accreditation, remains substantially undeveloped, creates a significant validity challenge for accreditation providers, policymakers and researchers. Achieving and maintaining an accreditation status requires a significant investment of resources, and for many organisations, the cost-effectiveness is debatable, including whether or not accreditation demonstrates a quantifiable improvement in healthcare delivery and outcomes [14]. Many countries are embarking on accreditation programs without any evidence about their effectiveness [5]. Nevertheless, without an empirically grounded, comprehensive evidence base for accreditation, the varying positive and negative views about accreditation will remain anecdotal and influenced by ideology or preferences [13]. Therefore, this is the first study that uses time series analysis over a 4-year period to demonstrate the impact of accreditation on quality measures. In addition, the study makes recommendations on the fundamental components of an accreditation program required to mitigate this effect and sustain improvement. It is argued that the implementation of standards combined with an external survey is no guarantee for continuous improvement. There needs to be a paradigm change from that of a snap-shot review to a continual assessment [37]. Accreditation can make a contribution to business improvement but if used incorrectly it can result in a bureaucratic system that is complex to sustain and engage staff. The study shows that while accreditation has residual benefit three years later, continuous survey readiness, frequent self-assessment, frequent external review and other continuous quality improvement methods are necessary to sustain the positive impact of accreditation.

## Abbreviations

JCI: Joint Commission International
TJC: The Joint Commission
HAAD: Health Authority-Abu Dhabi
US: United States
UAE: United Arab Emirates
CBC: Complete blood count
FMEA: Failure mode effects analysis
IPSG: International patient safety goals

## References

[1] Kohn L, Corrigan J, Donaldson M (1999). To err is human: Building a safer health system.

[2] Baker GR, Norton P (2003). Patient safety and healthcare error in the Canadian healthcare system: a systematic review and analysis of leading practices in Canada with reference to key initiatives elsewhere.

[3] Berwick D, Calkins D, McCannon C, Hackbarth AD (2006). The 100 000 lives campaign: setting a goal and a deadline for improving health care quality. JAMA.

[4] Braithwaite J, Westbrook M, Travaglia J, Iedema R, Mallock NA, Long D (2007). Are health systems changing in support of patient safety? A multi-methods evaluation of education, attitudes and practice. Int J Health Care Qual Assur.

[5] Shaw CD (2003). Evaluating Accreditation. Int J Qual Health Care.

[6] Greenfield D, Travaglia J, Braithwaite J, Pawsey M (2007). An analysis of the health sector accreditation literature. A report for the Australian Accreditation Research Network: examining future health care accreditation research.

[7] Griffith JR, Knutzen ST, Alexander JA (2002). Structural versus Outcomes Measures in Hospitals: A Comparison of Joint Commission and Medicare Outcomes Scores in Hospitals. Qual Manag Health Care.

[8] Salmon JW, Heavens J, Lombard C, Tavrow P, Heiby JR, Whittaker S (2003). Quality assurance project ii: a randomized controlled trial of a hospital accreditation programme with commentaries and foreword. S Afr Oper Res Results.

[9] Øvretveit J, Gustafson D (2003). Improving the quality of health care: Using research to inform quality programmes. BMJ.

[10] Miller MR, Pronovost P, Donithan M, Zeger S, Zhan C, Morlock L (2005). Relationship between Performance Measurement and Accreditation: Implications for Quality of Care and Patient Safety. Am J Med Qual.

[11] Braithwaite J, Westbrook J, Pawsey M, Greenfield D, Naylor J, Iedema R (2006). Study Protocol: A Prospective, Multi-Method, Multi-Disciplinary, Multi-Level, Collaborative, Social-Organisational Design for Researching Health Sector Accreditation. BMC Health Serv Res.

[12] James M, Hunt K (1996). Accreditation at What Cost?. J Manag Med.

[13] Greenfield D, Braithwaite J (2008). Health Sector Accreditation Research: A Systematic Review. International J Qual Health Care.

[14] Nicklin W, Dickson S (2009). The Value and Impact of Accreditation in Health Care: A Review of the Literature.

[15] Mannion R, Davies H, Marshall M (2005). Impact of star performance ratings in English acute hospital trusts. J Health Serv Res Pol.

[16] Chuang S, Inder K (2009). An effectiveness analysis of healthcare systems using a systems theoretic approach. BMC Health Serv Res.

[17] Walshe K (2007). Understanding what works–and why–in quality improvement: the need for theory-driven evaluation. Int J Qual Health Care.

[18] Greenfield D, Braithwaite J (2009). Developing the evidence base for accreditation of healthcare organisations: a call for transparency and innovation. Qual Saf Health Care.

[19] Joint Commission International. http://www.jcrinc.com/ accessed 17^th^ September 2013.

[20] Chandra A, Glickman SW, Ou FS, Peacock WF, McCord JK, Cairns CB (2009). An analysis of the association of society of chest pain centers accreditation to American college of cardiology/American heart association non-st-segment elevation myocardial infarction guideline adherence. Ann Emerg Med.

[21] El-Jardali F, Jamal D, Dimassi H, Ammar W, Tchaghchaghian V (2008). The Impact of Hospital Accreditation on Quality of Care: Perception of Lebanese Nurses. International J Qual Health Care.

[22] Sack C, Lütkes P, Günther W, Erbel R, Jöckel K, Holtmann G (2010). Challenging the Holy Grail of Hospital Accreditation: A Cross-sectional Study of Inpatient Satisfaction in the Field of Cardiology. BMC Health Serv Res.

[23] Bowling A (2002). Research Methods in Health: Investigating Health and Health Services.

[24] Chen J (2003). JCAHO accreditation and quality of care for acute myocardial infarction. Health Aff (Millwood).

[25] Cook TD, Campbell DT (1979). Quasi-experimentation. Design and analysis issues for field settings.

[26] Gillings D, Makuc D, Siegel E (1981). Analysis of interrupted time series mortality trends: an example to evaluate regionalized perinatal care. Am J Public Health.

[27] Wagner AK, Soumerai SB, Zhang F, Ross-Degnan D (2002). Segmented regression analysis of interrupted time series studies in medication use research. J Clin Pharm Ther.

[28] Lagarde M, Palmer N. The impact of user fees on access to health services in low- and middle-income countries. Cochrane Database of Systematic Reviews Issue 4. 2011, Art. No.: CD009094. DOI: 10.1002/14651858.CD009094. 10.1002/14651858.CD009094 PMC10025428 21491414

[29] Chatfield C (1989). The Analysis of Time Series: An Introduction.

[30] Dickey D, Fuller WA (1979). Distribution of the estimates for autoregressive time series with a unit root. J Am Stat Assoc.

[31] Yaffee R, McGee M (2000). Introduction to Time Series Analysis and Forecasting with Applications of SAS and SPSS.

[32] Scrivens E, Klein R, Steiner A (1995). Accreditation: what can we learn from the Anglophone model?. Health Policy.

[33] Braithwaite J, Greenfield D, Westbrook J, Pawsey M, Westbrook M, Gibberd R (2010). Health Service Accreditation as a predictor of clinical and organisational performance: a blinded, random, stratified study. Qual Saf Health Care.

[34] Chuang S, Howley PP, Hancock S (2013). Using clinical indicators to facilitate quality improvement via the accreditation process: an adaptive study into the control relationship. Int J Qual Health Care.

[35] Piotrowski M (2005). Are you ready for unannounced surveys?. Biomed Instrum Technol.

[36] Øvretveit J (2001). Quality evaluation and indicator comparison in health care. Int J Health Plann Mgmt.

[37] Devkaran S, O’Farrell PN. The impact of hospital accreditation on clinical documentation compliance: a life cycle explanation using interrupted time series analysis. BMJ Open 2014. 4:8 e005240 doi:10.1136/bmjopen-2014-005240. 10.1136/bmjopen-2014-005240 PMC4127940 25095876

